# Supplementary material for: Patterns of Intron Gain and Loss in Fungi
Source: PLoS Biol. 2004 Nov 30;2(12):e422. doi: 10.1371/journal.pbio.0020422 (PMC532390; doi:10.1371/journal.pbio.0020422)
Supplement: Table S1 — Also available at http://genes.mit.edu/NielsenEtAl/. (4.3 MB ZIP). [file pbio.0020422.st001.zip › NielsenEtAl/html/273.html]

AN5230.1.NCU03211.1.MG03679.1.FG00670.1


```
 CLUSTAL W (1.82) Multiple Sequence Alignments - Introns Inserted


Sequence 1: MG03679.1	325 aa
Sequence 2: FG00670.1	306 aa
Sequence 3: NCU03211.1	361 aa
Sequence 4: AN5230.1	310 aa
Alignment Length: 367 aa
Number Identitical Residues: 64 aa
Alignment Score (without introns) 4565


MG03679.1 	MGWYERIFG-GKAAD---PLEKLDPKLREFLDKESPVKLQKAQEEKQRREQQQKQAD---
NCU03211.1	MGWFDGWFGNSSSSDESDPLGKLDPKLREFLARESPVKYTTQQEQEQQQQQQQQQQQQKP
FG00670.1 	MGWISSILGTDKSAD---PLAKLDPSLRDFLEKESPLKYPANQP----------------
AN5230.1  	MGWFWGSNN----ED---PVKKLDPGLREYLENEAPKKYVPASQ----------------
          	***     .     *   *: **** **::* .*:* *    .                 

MG03679.1 	------VKAAAAAVQKTESHSS---SSTSADKASGVPKESLYQDGRYAHLWKNYRPLSEI
NCU03211.1	RAKQYLASAPAAPVPTAKVPATEQPQQEQQQQQPAVPRASLYQDGRYAHLWKTYRPQYEI
FG00670.1 	------AHPAQAVQKPSNTNVT---VAETQGQKPAAPSASLYKDGRYAHLWKSYRPLAEI
AN5230.1  	-------SPPAAEEKASPSTTS---SDDASSASPAVPSASLFPDGRYAHLWKTYKPPSPG
          	        .. *    :    :           ...*  **: *********.*:*    

MG03679.1 	EGESKTDHEKLQDVLEAYKSRKEQIGRAALENCALEQFEWRDCMNNG-SWEAKLTMCSKQ
NCU03211.1	EAETKTDHEKLMDVLEGFKERKAMIGKAALENCAEEQLDWNNCMKHG-SWADRATMCRDT
FG00670.1 	EAETASDHEKLMSVLEAYKERKEAIGKAALENCAEYQEEWVNCMKHG-SWEDQIQMCRNQ
AN5230.1  	EVG--PETTGVPSLKAHSKQRQAMVKRAAMENCSIENEIYHLCLKSPPTMSDRLTACQDK
          	*    .:   : .:    *.*:  : :**:***:  :  :  *::  .:   :   * . 

MG03679.1 	VKKFERCYSMQA0RLLKTLGYLSTYERPPSVDEDIQMHADALYHRMIEQEEAIEKAKAEK
NCU03211.1	VRKFERCYNMQS0KLLKALGYLQTYGRSPEVDEDIQMHADALYHRMLDQEAEIEKCKAEG
FG00670.1 	VRSFERCYLMQS0RFLRALGHGSVVGRSPEIEDDIQMHADALYQRMIKHEEAVEKAKAEG
AN5230.1  	NKVFTRCFTTQS0KFLQALGYASAHQWDDEREERIQLHADKLYHEMLDYEKQVEEAREAG
          	 : * **:  *: ::*::**: ..     . :: **:*** **:.*:. *  :*:.:   

MG03679.1 	KPLPQFAPLLIKPII-----------------------AAEDEKFQLSQEQRKEMN--QR
NCU03211.1	RPIPAFPSLFENSTYKVPVYPTKTSAAPQSTSAAGAGTEPGKDPYEPSQAIVESWQ--EE
FG00670.1 	LPVPVFQSKLPK---------------------------AKDDSVVPTEELQQQWK--EQ
AN5230.1  	REPPPLTSLFNPQAKPIER----------------ASVGPAELEIPGGEPIPEGFTPSKP
          	   * : . :                         :.  . .      :   :   .:: 

MG03679.1 	LAKIPEAERAMEEEAIKAEYRAKAEVGHKIQDLWKENEKDREKRKKEGQETVLDKVSGMW
NCU03211.1	LAKLPELERAAQEEALRAEHRAKAIMAKNLQELWNEQSKEREQRRMEGRETIGDKIKRVL
FG00670.1 	LDKLPAEERAVEEAALRADLQVKAEVAGNVQKIWDTQAEQRKQRQAEGNSTFGDYMASLF
AN5230.1  	LERLTPHERELEIRAYYQQLEEQKAYVKGMLPVIRSHDEARDKRREKLVGWFGETVGNWM
          	* ::.  **  :  *   : . :      :  :   : : *.:*: :    . : :    

MG03679.1 	KGSGGNDK
NCU03211.1	GTPNY---
FG00670.1 	GKSGK---
AN5230.1  	TR------
          	
```
